# Human Movement Recognition Based on 3D Point Cloud Spatiotemporal Information from Millimeter-Wave Radar

**DOI:** 10.3390/s23239430

**Published:** 2023-11-27

**Authors:** Xiaochao Dang, Peng Jin, Zhanjun Hao, Wenze Ke, Han Deng, Li Wang

**Affiliations:** College of Computer Science & Engineering, Northwest Normal University, Lanzhou 730070, China; 2021222246@nwnu.edu.cn (P.J.); haozhj@nwnu.edu.cn (Z.H.); 2021222210@nwnu.edu.cn (W.K.); 2021222276@nwnu.edu.cn (H.D.); 2021222195@nwnu.edu.cn (L.W.)

**Keywords:** 3D point cloud, millimeter-wave radar, human movement, neural network

## Abstract

Human movement recognition is the use of perceptual technology to collect some of the limb or body movements presented. This practice involves the use of wireless signals, processing, and classification to identify some of the regular movements of the human body. It has a wide range of application prospects, including in intelligent pensions, remote health monitoring, and child supervision. Among the traditional human movement recognition methods, the widely used ones are video image-based recognition technology and Wi-Fi-based recognition technology. However, in some dim and imperfect weather environments, it is not easy to maintain a high performance and recognition rate for human movement recognition using video images. There is the problem of a low recognition degree for Wi-Fi recognition of human movement in the case of a complex environment. Most of the previous research on human movement recognition is based on LiDAR perception technology. LiDAR scanning using a three-dimensional static point cloud can only present the point cloud characteristics of static objects; it struggles to reflect all the characteristics of moving objects. In addition, due to its consideration of privacy and security issues, the dynamic millimeter-wave radar point cloud used in the previous study on the existing problems of human body movement recognition performance is better, with the recognition of human movement characteristics in non-line-of-sight situations as well as better protection of people’s privacy. In this paper, we propose a human motion feature recognition system (PNHM) based on spatiotemporal information of the 3D point cloud of millimeter-wave radar, design a neural network based on the network PointNet++ in order to effectively recognize human motion features, and study four human motions based on the threshold method. The data set of the four movements of the human body at two angles in two experimental environments was constructed. This paper compares four standard mainstream 3D point cloud human action recognition models for the system. The experimental results show that the recognition accuracy of the human body’s when walking upright can reach 94%, the recognition accuracy when moving from squatting to standing can reach 84%, that when moving from standing to sitting can reach 87%, and the recognition accuracy of falling can reach 93%.

## 1. Introduction

Nowadays, in the context of the rise of AI, IOT, and 5G, there are various means and ways of human–computer interaction that play significant roles in different fields [1], making people’s lifestyles change from unintelligent to intelligent. Both contact and non-contact sensing technologies have been applied in smart homes, health detection, intelligent transportation, automatic driving, smart medical care, etc. These two sensing methods are mainly applied through wearable devices [2] and wireless sensing-based technology for intelligent sensing. Among these methods, wearing a smart bracelet to detect heart rate, sleep quality, and other indicators is a typical application of wearable technology. Furthermore, the non-contact sensing technologies or devices based on wireless sensing include computer vision, Bluetooth, ultrasonics, Wi-Fi, LoRa, and so on. For example, in the application of the intelligent Internet of Things and smart homes, there are intelligent home appliance controls based on voice recognition and heart rate detection. These use wearable recognition technology in the form of a bracelet [2,3]; however, wearable device technology brings a lot of inconvenience and poses security risks to people. As people pay more attention to the security of their personal information and privacy, more convenient and secure human–computer interaction methods have emerged, such as iris recognition, face recognition, and palm print recognition [4,5,6]. Human–computer interaction based on human physiological features does not require users to carry additional tools such as wearable devices, smartphones, and identity tokens. Intelligent sensing is carried out more conveniently and safely in the context of identification, health monitoring, and smart homes, which can protect people’s data information and personal privacy.

However, physiologically based human–computer interaction, as shown in previous studies, also has many drawbacks; for example, iris recognition, face recognition, palm print recognition technologies, and traditional computer vision technologies all have a considerable risk of information leakage. For example, these technologies have the risk of being maliciously invaded, leading to the breach of people’s privacy and leakage of important data, which in turn leads to the theft of people’s physiological and identity information data. Palm print recognition, a type of contact recognition technology, also has the risk of disease transmission [7,8,9]. Therefore, human–computer interactions based on contactless technology and more secure human–computer interactions that protect users’ privacy have emerged.

Therefore, researchers try to use Wi-Fi for wireless perception and conduct much research on human–computer interactions. Some researchers have proposed the use of Wi-Fi perception technology in the field of human motion perception, and this new human–computer interaction mode can better protect people’s identity information and sensitive data compared with computer vision and other perceptual technologies. The most important thing is that, at low light levels and in dim conditions, Wi-Fi perception technology will perform more effectively in the recognition of human movement because each person’s unique action behavior, body type, and other body characteristic data will show different and special channel state information (CSI) under Wi-Fi signals. This information can be used to identify different people’s action behavior [10] and then identify their identity or some human movements. In [11], researchers proposed using Wi-Fi to perceive people’s gait and combined the LSTM neural network with Wi-Fi perception technology to design a very efficient six-layer gait recognition method. Another researcher designed a deep learning model CNN based on the convolutional layer combined with LSTM networks, and then realized the recognition of different people using Wi-Fi. However, Wi-Fi signals are highly susceptible to interference from other wireless signals, and Wi-Fi sensing technology requires a transmitter to transmit Wi-Fi signals and a receiver to receive Wi-Fi signals in terms of its deployment conditions, enabling the signal area between the transmitter and receiver to be effectively sensed. Wi-Fi signals will also decay with the increase in distance [12]. This will limit the perception area, which means that many broad application scenarios cannot take advantage of perception. The recognition area of computer vision is wider compared to Wi-Fi, and some good cameras can even reach a radius of several kilometers. Due to the limitation of perceptual distance and range, Wi-Fi is not the best intelligent perception mode. In addition, the Wi-Fi perception mode will be affected by its bandwidth, and it cannot identify the human body in some complex human–computer interaction scenarios where multiple people coexist.

Because millimeter-wave radar sensing technology does not have the above problems, it has attracted large amounts of attention in recent years. Nowadays, with the development of 5G high-speed rates and intelligent Internet of Things, millimeter-wave radar is more widely used in automatic driving and the behavioral perception of indoor human movement. In recent years, some researchers have begun to study the application of millimeter-wave radar signals in human movement recognition. Millimeter-wave radar signals have very high bandwidths, which can be as high as 4 GHZ. This attribute is mainly used in the frequency modulation signal to narrow the pulse on the target of the fine-grained characteristics of the acquisition. As such, the millimeter-wave radar signal has a high fine-grained spatial resolution, as well as precise target imaging, for more than one target, making detection and identification more accessible. Whether applied to walls or fog, dust, etc., this technique has strong penetration ability and can work all day long, meaning there is less interference. Moreover, millimeter-wave radar does not have the severe problem of leaking people’s privacy and important information like computer vision, and it does not affect the sensitivity of capturing point clouds due to unfavorable weather conditions and turbid air like LiDAR [13]. As such, researchers are keen to use millimeter-wave radar in the field of human movement recognition.

Millimeter-wave radar devices have comprehensive advantages for human movement recognition compared to other sensing devices due to their smaller size, lower cost, high penetration capability, and high spatial resolution. Some researchers have provided an overview of neural network-based human movement classification methods [14]. Another study verified the feasibility of convolutional neural networks for classifying human actions based on time and distance images [15]. The combination of feature vectors has also been utilized to classify nine sets of actions [16]. Some scholars also applied unsupervised machine learning methods to recognize human walking and standing actions [16].

To sum up, in order to solve the above problems, this paper proposes to use millimeter-wave radar to collect the dynamic point cloud data of human movements for the classification and recognition of human movements. Compared with some previous studies, the contributions of this paper are mainly as follows:

(1) This paper proposes a neural network model PNHM based on PointNet ++ to realize dynamic point cloud processing. The model slices the de-noised dynamic point cloud and inputs the attribute information of the point cloud data of each frame into the neural network for training and classification. The model can extract features of the three-dimensional point cloud’s time, space, speed, and other information in order to integrate local features with global features.
(2) This paper proposes to apply dynamic point clouds to several types of essential human motion recognition: walking upright, standing to sit down, standing to squat, and falling. In addition, the collection and recognition of human motion point cloud data is carried out in five experimental scenarios, which have high accuracy and robustness in multiple scenarios.
(3) In the model, we add the placement of the radar is placed at a distance of 2.5 m from the ground. The threshold method is used to identify the difficulty of identifying similar actions by sitting down and squatting. The speed and height thresholds of the point cloud are used to perform identification again, overcoming the influence of height difference and improving trobustness.

## 2. Related Work

Human action recognition has essential applications in wise aging, child safety monitoring, health detection, and human–computer interactions, and researchers have attempted to apply different perceptual modalities to these fields. Furthermore, previous studies have applied visual images and various wireless perceptual modalities to human action recognition. For example, some researchers have used supervised learning methods to train human behavior recognition models in organizing behavioral data samples based on video streams [17,18]. This technology performs very well in human action recognition accuracy but, under the high requirements of privacy and information security, video images can have many limitations. For instance, if the camera is maliciously attacked and hijacked, the inclusion of factual image information can leak personal privacy and essential information, and also induce poor robustness. In many adverse conditions, such as rain, snow, haze, and dusty environments, video images do not perform well in the recognition mode, especially in the traffic police action recognition in the automatic driving scenario. As such, computer vision perception is very demanding on the environment.

Therefore, researchers have applied various electromagnetic wave device-based perception techniques to human movement perception. There is a large amount of research work in human movement recognition based on the perception of electromagnetic methods. The common ones are Wi-Fi perception, acoustic wave perception, etc. Since Wi-Fi devices can be found everywhere in life, the research of human movement recognition based on Wi-Fi devices is the most researched topic. The principle of recognition based on Wi-Fi devices is based on the fact that each person’s body behavior is different, leading to the channel state information (CSI) changing significantly and generating different features. These features are trained via machine learning to classify and recognize human actions [18]. However, Wi-Fi sensing technology cannot distinguish the signals of multiple targets well enough for the classification and recognition in human action recognition scenarios with multiple people. As such, there will be great difficulties in human action recognition in coexistence scenarios with multiple people. Wi-Fi is less robust to human behavioral action recognition in different environments, and it cannot perform well in different environments.

In a recent study, researchers used millimeter-wave radar for gait recognition of micro-Doppler signal features generated by the human gait while walking [19] and constructed a CNN-based neural network model to extract as well as classify gait micro-Doppler features. Experiments on five targets of gait data for classification and recognition found the effect of the method’s performance in the error rate of 21.5%. mHomeGes designed a lightweight neural network mGesNet. Point cloud features and micro-Doppler features were fused to extract the gesture features for the elimination of the multipath effect, and the authors designed a hidden Markov model-based HMM-VM mechanism for continuous gesture recognition in the complexity of the multipath effect. The recognition rate of mHomeGes for gestures can reach 95.3%, even in complex and changing environments [20]. Zhou Anfu’s team at Beijing University of Posts and Telecommunications designed a neural network, mmGaitNet, to recognize different people’s gaits by fusing multiple features using the point cloud data formed by different people’s gaits in multiple- and single-person scenarios [21].

Moreover, these studies are able to recognize single or relatively straightforward human actions, but far less research has been conducted about complex human actions and the different aspects of complex scenes. Therefore, in this paper, we utilize millimeter-wave radar 3D point cloud to conduct experiments in five scenes under two experimental environments to recognize four basic human actions [22,23,24]. In the experiment, the interference of the environment and dynamic objects cannot be avoided, and only by obtaining the point cloud that excludes the interference of the environment can the recognition rate of the human body action be improved. Thus, in this paper, the 3D point cloud data are denoised. The point cloud under the spatiotemporal domain is clustered to obtain the point cloud of the human body action based on the distance metric. The PNHM method is designed based on PointNet++, and the point cloud data from the beginning to the end of the point cloud data are used for a single experimental subject; hence, no segmentation of the point cloud data is required. In order to obtain better accuracy and reduce the impact on bandwidth resources and time, the dynamic point cloud is sliced. Experimental comparisons are performed in five scenarios, and unknown actions are predicted as unknown experimental subject’s actions are recognized. This paper compares mainstream point cloud recognition algorithms from recent research with the PNHM method.

## 3. 3D Point Cloud Generation for Millimeter-Wave Radar

**Figure 1:**
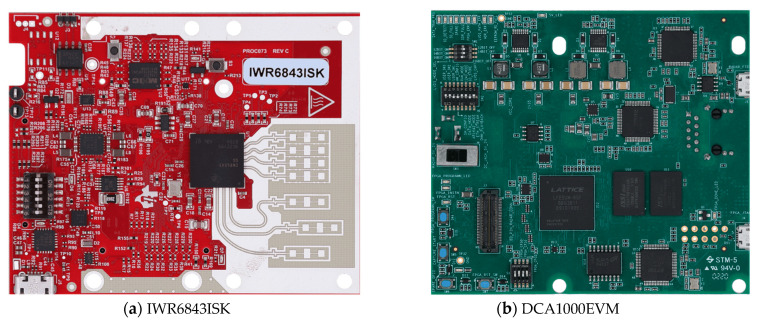
IWR6843ISK and DCA1000EVM.

In this paper, IWR6843ISK millimeter-wave radar with DCA1000EVM is used for point cloud data acquisition, as shown in Figure 1a,b. The millimeter-wave radar consists of three transmitting antennas, four receiving antennas, a built-in phase-locked loop (PLL), and an analog-to-digital converter (ADC). The radar integrates a digital signal processor with a maximum bandwidth of 4 GHz and a distance resolution of 3.75 cm. The two devices are connected to a computer through a serial port, and the radar’s actual frequency is set to 60 GHz. Each time, the ADC1000EVM samples 1000 frames. The raw data of the point cloud are collected, processed, and trained by processing the data in Matlab and PyTorch environments using Linux.

The millimeter-wave radar device transmits a continuous FM signal wave by transmitting the radar signal to the target moving object. The IF signal reflected from the target human body is obtained. After systematic processing, it is presented in this paper in the form of a 3D point cloud. The 3D point cloud signal contains the 3D coordinates of the moving human body, the time information, and the information such as the signal-to-noise ratio. This paper uses an IWR6843 device three-transmitter, four-receiver millimeter-wave radar equipment. Its transmission frequency follows the time and has a linear increase, showing a sinusoidal form of the signal. The radar signal emitted by the millimeter-wave radar IWR6843 is represented as [25]:

(1) $$s_{T}(t)=A_{T}\cos2\pi [f_{c}t+\int_{0}^{t}f_{T}(\tau )d\tau ]$$

The radar signals it receives are indicated as:

(2) $$s_{R}(t)=A_{R}\cos2\pi [f_{c}(t-\Delta t_{d})+\int_{0}^{t}f_{R}(\tau )d\tau ]$$

In Equation (1), $s_{T}(t)$ denotes the radar-transmitting signal, $A_{T}$ denotes the intensity of the radar-transmitting signal, $f_{c}$ denotes the center frequency of the carrier wave, and $f_{T}$ denotes the frequency of signal transmission. In Equation (2), $s_{R}(t)$ represents the radar-received signal, and $A_{R}$ represents the intensity of the radar-received signal.

The core data in this paper are obtained by acquiring information in multiple dimensions of the 3D point cloud, to which the spatial coordinate information (x,y,z) of the experimental object and the velocity and altitude attributes are highly critical and essential. The millimeter-wave radar transmits signals to the experimental object and returns $\omega =\frac{2\pi \Delta d}{\lambda }$ to the radar-receiving antenna. The distance between the antennas is d for the target azimuth θ, meaning that the path difference is Δd=dsinθ, which is denoted as [26]:

(3) $$\omega =\frac{2\pi \Delta d}{\lambda }$$

(4) $$\theta =\sin^{-1}(\frac{\lambda \omega }{2\pi d})$$

In Equation (3), λ is the wavelength of the radar signal. The phase difference between the signals on the antennas of the neighboring received signals can be found in ω, and Equation (4) calculates the azimuth angle θ of the experimental object.

This paper involves the information of velocity, acceleration and angle of three-dimensional point cloud in the three directions of *x*, *y* and *z*. The elevation angle of the experimental object can be calculated according to the height difference of the transmitting antenna, and the three-dimensional spatial coordinate information of the testing object can be calculated using the radar’s azimuth, elevation angle, and the transmission distance, as expressed by the following formula [26]:

(5) $$\left\{\begin{array}{c} x=r\cos(\varphi )\sin(\theta )\\ y=r\cos(\varphi )\cos(\theta )\\ z=r\sin(\varphi ) \end{array}\right.$$

It is also necessary to calculate the meridional velocity of the experimental subject; the time interval between two consecutive chirps is $T_{c}$. The displacement of the experimental subject is Δd, and the phase difference ω can be obtained from Equation (3). Then, the radial velocity of the experimental subject is expressed as [26]:

(6) $$\nu =\frac{\lambda \omega }{4\pi T_{c}}$$

IWR6843 millimeter-wave radar process the received signal and transmit the signal for mixing to obtain the IF signal. If the distance between the experimental object and the radar before is R, the electromagnetic wave speed is c, and the IF signal is expressed as [27]:

(7) $$S_{IF}=e^{i2\pi (K\frac{2R}{c}t)}=e^{i2\pi (f_{IF}t)}$$

For a single experimental subject, the IF signal is a single-frequency signal; after Fourier transform (FFT), the IF can be obtained by finding the location of the peaks appearing in a single IF signal. According to $f_{IF}=K\frac{2R}{c}$, the distance can be expressed as [27]:

(8) $$R=\frac{f_{IF}c}{2K}$$

**Figure 2:**
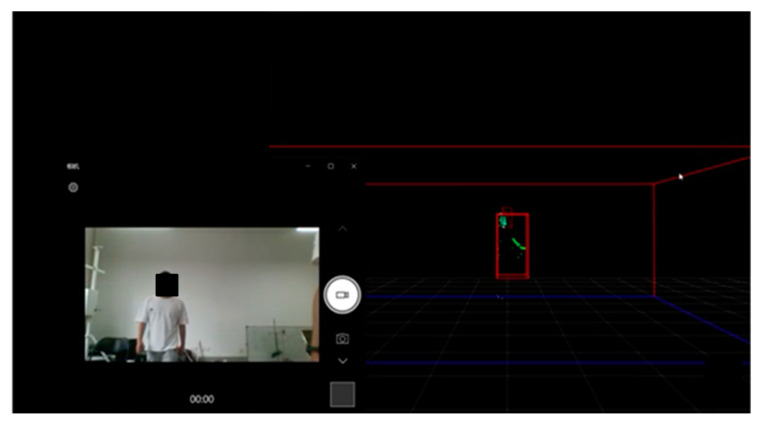
Raw data for 3D point cloud formation.

The point cloud generation tested in the indoor environment is shown in Figure 2. The following pictures visually record the dynamic point cloud generated by the experimental subject in the movement process through video recording, which ends when the point cloud data reaches 1000 frames. Each frame contains the point cloud of 0–64 human body movement scattering points. Different movements will have different regularity of operation. Accordingly, the movement characteristics of the human body’s various scattering points will also have regularity of changes, and the acquired 3D point cloud maintains continuity in time and space. By processing the dynamic point cloud, we can derive the features of different movements and use a neural network to extract the features of the data to realize the recognition of different movements. It can be seen that the raw point cloud data, labeled in green, are presented in real time as the experimental subject presents its movements in 3D space.

## 4. PNHM Method Design

**Figure 3:**
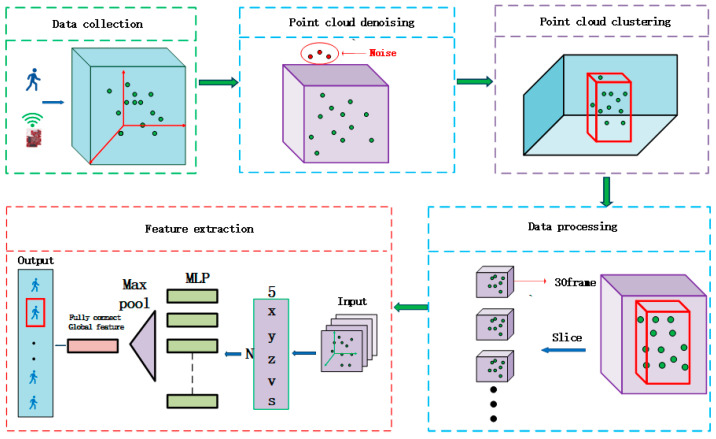
Flowchart of the PNHM method.

Millimeter-wave radar uses the physical characteristics of human motion for motion recognition. Different movements of the human body in the millimeter-wave radar IWR6843’s FMCW continuous wave signal present different 3D point cloud features due to the different radar reflection signals presenting a significant difference in the 3D point cloud information. As such, in this paper, we have designed the PNHM (PointNet++ Human Motion) method, which can be used to extract and categorize the features of the point cloud data of these movements to realize the recognition of the human body movements, as shown in Figure 3.

The system is divided into five steps. (1) 3D point cloud data acquisition: the millimeter-wave radar IWR6843 transmits FM signals, and the moving human body reflects the radar signals to the radar and generates 3D point cloud data streams over time in real-time. The acquisition process produces unavoidable noise due to the experimental environment and the means of data acquisition. (2) Point cloud denoising: this module uses the clustering-based denoising algorithm DBSCAN. The core idea of the method is to use the 3D point cloud spatial coordinates and attribute features to different categories because the noise is isolated or has a minimal density of points and will not be clustered with other points as a category, meaning that the clustering of some of the lowest number of points in the category is reversed to achieve the purpose of denoising. (3) Point cloud clustering: this module mainly gathers the sparse 3D point clouds in an experiment into a cluster, which is convenient for calculating the spatial properties of the point clouds formed by some actions. (4) Data processing: after processing in module (3), the point cloud data are dynamic 3D point cloud data, and the number of data frames produced by each action is different. In order to facilitate the unified input data in module (5), in two experimental environments, each kind of human action point cloud data is cut into 15, 30, 45, 60, 75, and 90 frames for training, respectively. The results show that, when the 3D point cloud is cut into 30 frames, it yields the best classification effect, and so this module unifies all data according to the 30 frames to be cut as the input data of the module (5). (5) Feature extraction: the cut point cloud data in module (4) are trained and tested according to 3:1; the five attributes of the point cloud are input into the point cloud features, and the local and global features of the point cloud are fused. In order to improve the accuracy of the experimental results and the experimental comparison effect, in this paper, the radar is placed on the front or side of the human body and at a height of 2.5 m from the ground. Experiments are also set up under various angles to predict the unknown human body movements, where the red box represents the predicted action classification type, and compare the accuracy of the point cloud data of the movements of the unknown experimental objects with the accuracy of the known experimental object point cloud data obtained in this paper. In conjunction with the recent research, the mainstream 3D point cloud recognition algorithms are used to recognize the human actions in this paper, and a large number of comparison experiments are conducted to obtain the human action recognition rates in five scenarios under different algorithms.

**Figure 4:**
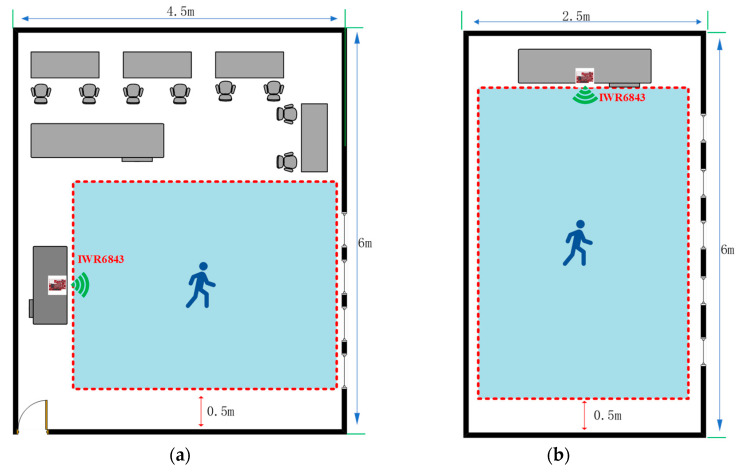
Data collection in laboratory and corridor scenarios. (**a**) Laboratory data collection; (**b**) corridor data collection.

**Figure 5:**
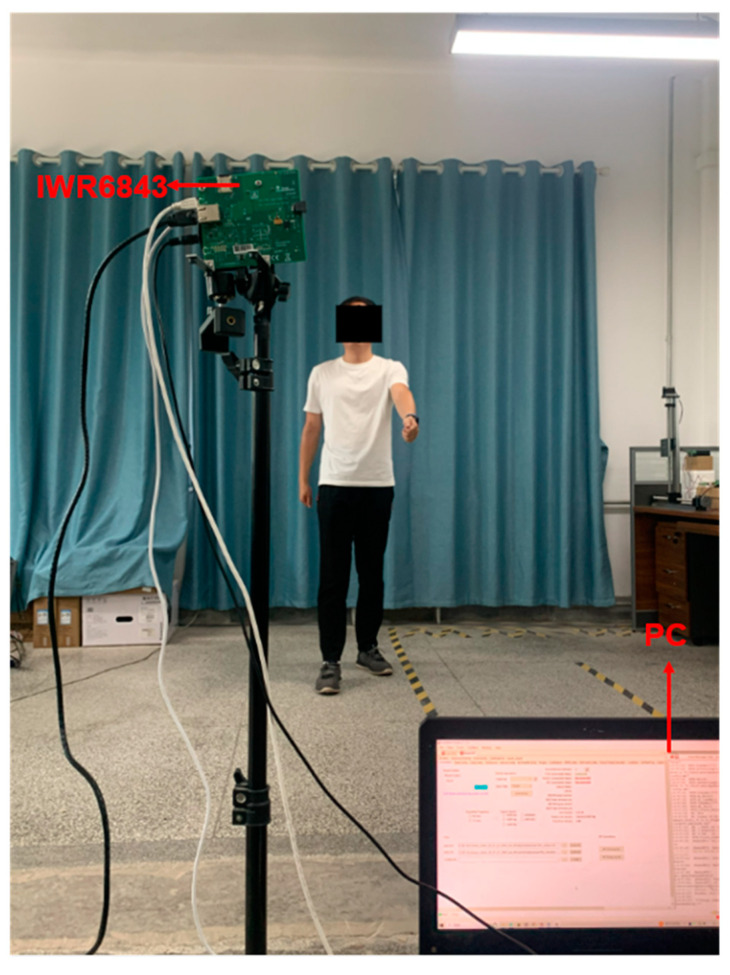
Laboratory scene and equipment.

In this paper, we design methods to collect experimental data in two experimental environments, the data collection laboratory as well as the corridor, and the experimental environment and equipment are shown in Figure 4a,b. Because of the consideration that, if the human body movement follows the random route, then the 3D point cloud presented will become more complicated in space, which is not conducive to obtaining the point cloud rule characteristics of human body movement in order to better present the characteristics of human body movement using the point cloud, two experimental environments are designed to make the human movement route and the center of radar transmitting signals present 0° and 30° angles for the collection of human body movement point cloud data, as shown in Figure 5. And in order to present the diversity of 3D point cloud data in the experiment, we considered the distances, angles, different weights, different heights, different genders, different ages of the experimental subjects in the actual scene, etc., under the two angles. The factors related to the experimental subjects, radar heights, and distances between the experimental subjects and the radar present a variety of diversity, and so we also collected data for these. In this paper, four boys and four girls were selected as experimental subjects and the eight experimental subjects had different heights and weights. The millimeter-wave radar IWR6843 BOOST is composed of three transmitting antennas and four receiving antennas, and each frame of the data obtained is theoretically a 64-point cloud. Data acquisition conducted indoors is susceptible to the interference of some dynamic objects and static objects. For example, human body movement is caused by the shaking of curtains and the shaking of fan blades. Additionally, the walls, tables, and chairs can affect the radar for the movement of the human body and produce the wrong measurements, etc. All of these factors will generate some human body action point cloud outside the noise, resulting in the generation of scattered and sparsely distributed 3D point cloud data. As such, in this paper, we must first of all perform point cloud data denoising, using DBSCAN algorithm to improve the accuracy and quality of the point cloud data for better feature classification.

**Figure 6:**
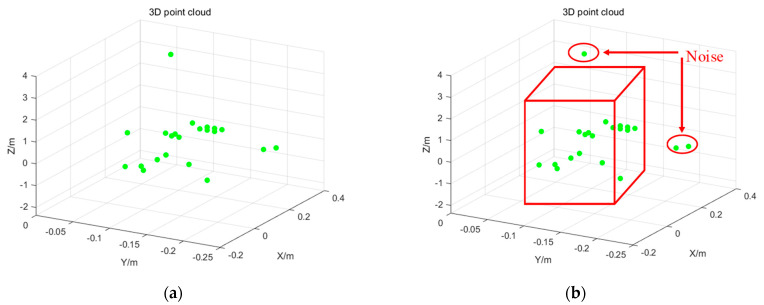
Clustering and denoising of single person action point cloud in 3D space. (**a**) Original 3D spatial point cloud; (**b**) point cloud clustering denoising.

This paper adopts the clustering-based point cloud denoising method because the surrounding environment is often isolated by the noise, or the density is smaller than the density of the point cloud formed via human body movement. This interfering noise does not form a clustering category with the human body movement point cloud, and so this paper uses the three-dimensional space according to the spatial attributes of the point cloud and by setting the velocity threshold to discriminate the static point cloud using the original point cloud data shown in Figure 6a. The point cloud is clustered for containment in a rectangular box to form a cloud. The point cloud outside the cloud is recognized as noise to be removed, leaving the point cloud inside the cloud as the point cloud formed by the human body movement, as shown in Figure 6b.

In the five experimental scenarios in this paper, because most of the dynamic noise formed by the interference is a certain distance away from the experimental object-formed point cloud, this paper adopts an algorithm based on the density of the DBSCAN spatial clustering, using the DBSCAN algorithm for the clustering of the core idea: the density of the point cloud of the density of the reachable distance of the relationship, formed by the maximum density of the set of samples connected to the clustering, is a cluster. The technique works as follows. First, it is necessary to choose from the data set of the point cloud to select arbitrary point cloud data and a point from the edge of the point. Then, it is necessary to perform re-point selection, with a as the center, to find out the of density accessible data object points, and by adjusting the radius of the domain Eps and the number of domain data object, the threshold minimum number of points MinPts parameter value, the formation of a cluster, the distance between the two-point cloud are lowered down to the values of Eps. The 3D point cloud (x,y,z) is the position of the localized part of the body of the experimental object movement; the clustering algorithm is a standard evaluation method operating through the contour coefficient. In the Formula (9), a(i) represents the average distance between the sample point i and the other sample points, and when its value is smaller than that of the sample point it will be clustered into the cluster, where b(i) represents the sample point i and the dissimilarity between the cluster, and where the average distance between the sample point i and all the sample points prior to the cluster is bij, bi=min(bi1,bi2,bi3,…,bik).

(9) $$s(i)=\frac{b(i)-a(i)}{\max\{a(i),b(i)\}}=\left\{\begin{array}{c} 1-\frac{a(i)}{b(i)},a(i)<b(i)\\ 0,a(i)=b(i)\\ \frac{b(i)}{a(i)}-1,a(i)>b(i) \end{array}\right.$$

When s(i) [28,29,30,31,32] is approximately 0, it means that sample point i is on the boundary of the cluster; when s(i) is approximately 1, it means that it is reasonable for sample point i to be in the cluster; and when it is approximately −1, it means that sample point i should not be clustered into the cluster.

In this paper, we introduced the fact that the point cloud is denoised and clustered before high-quality point cloud data are obtained due to several groups of actions being performed across different periods, such as moving from standing to sitting and performing upright walking. These two actions, performed in one time period, show key differences, with upright walking being an arm-swinging and repetitive leg-walking motion and lasting for a shorter duration relative to the time standing. When collecting different actions, different frame rates are set for the IWR6843 BOOST, with 5 frames per second for walking, 10 frames per second for standing to sitting or sitting to standing, 15 frames per second for standing to crouching or crouching to standing, and 20 frames per second for falling, which facilitates the unified point cloud data input with neural networks.

We collected point cloud data for several sets of fundamental movements of the human body. After denoising the point cloud to effectively extract the information features of 3D point cloud data in multiple dimensions of time and space, we designed a neural network PNHM based on PointNet++ and shown in this paper. In the previous research on the processing of point cloud, the first step involved segmenting the point cloud, and only then could the obtained target point cloud be processed for classification. In this paper, we collected 3D point cloud data from the movements of a single subject and identified and classified the overall movements of the subject’s whole body after denoising the point cloud data produced by the human body’s movements and the surrounding environment. We obtained the point cloud of the human body’s movements, which did not contain other point clouds in the experimental environment and did not need to be segmented. The PNHM method adopts a multilevel feature extraction structure, in which some 3D point clouds are selected as the center of the input point clouds generated by human body movements, and then some points around these points are used to form a region. Each region is used as a PNHM input once to obtain the spatiotemporal information features of the point clouds within these regions, and then the region is enlarged around the center points, and then the enlarged region is selected as an input to obtain the spatiotemporal information features of the point clouds within the larger region. After that, the region is expanded around multiple centroids, and the expanded region is selected again as an input to obtain the point cloud spatiotemporal information features of this larger region. The above process is continuously iterated to derive all the regions of the 3D point cloud by taking more significant regions as inputs. The point cloud spatiotemporal information features of the more significant regions are obtained. Then, many local and global features are obtained, before finally the classification is carried out based on global features.

**Figure 7:**
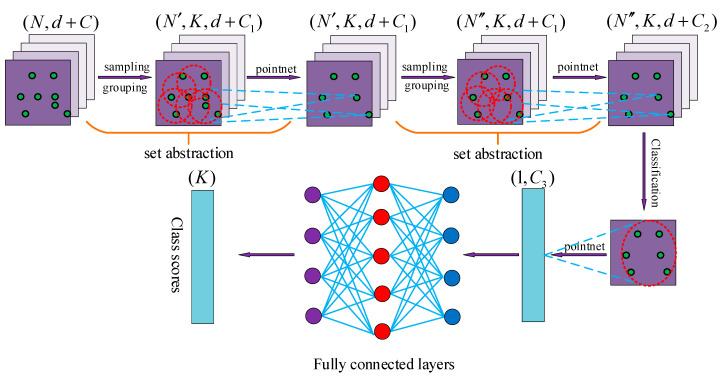
PNHM neural network structure.

The neural network structure of the PNHM method designed in this paper is shown in Figure 7. The PNHM method can be summarized as an encoder–decoder structure, in which the encoder part of the downsampling process is a hierarchical point set abstraction layer. The hierarchy is composed of multiple set abstraction structures, and the number K of sampling points and the radius R of the sampling of each set abstraction module are different. Moreover, K and R will increase with each additional layer so that each set abstraction can derive a different scale of local point cloud features, and the global features of the point cloud are obtained after many set abstraction outputs. The green dots represent the 3D point cloud, the red round box is a sampling process, and the blue dotted line represents a result after sampling. The dots of various colors in the final fully connected layer represent the inputs and outputs.

**Figure 8:**
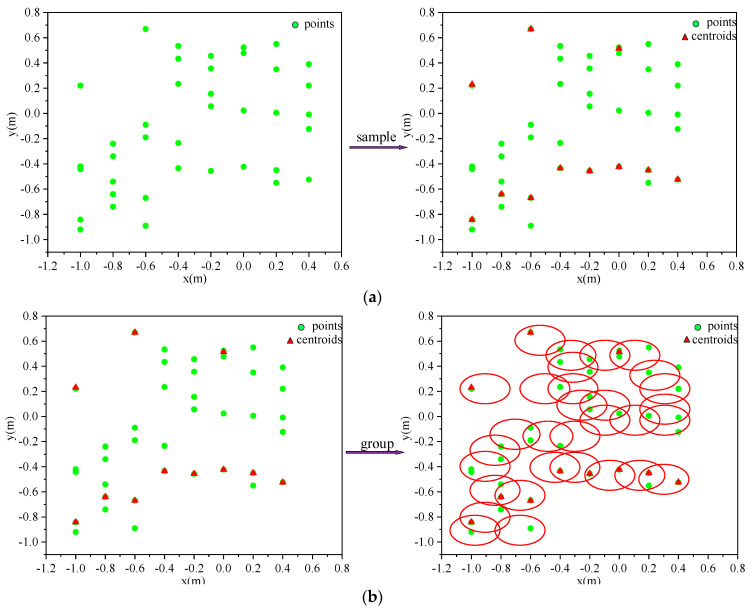
Point cloud sampling and grouping process. (**a**) Furthest point sampling process; (**b**) sample grouping process.

The sampling layer uses farthest point sampling to downsample the point cloud, as shown in Figure 8a. After a set abstraction, the point set size N can be changed to a smaller size $N^{\prime }$, which can make the distance between the sampled point clouds as large as possible, and the result of downsampling become more uniform. Farthest point sampling is realized by randomly selecting a point cloud as the initial point, then calculating the distance between the points in the unsampled point set and the sampled point set, adding the point with the most significant distance to the sampled point set, and then re-calculating and iterating in a loop until the target number of sampled point clouds are obtained, which will provide excellent coverage of all the sampled point clouds. The input to the grouping layer is the set of points of N∗(d+C), and the output is $N^{\prime }*K*(d+C)$. N represents the number of point clouds in the input, d represents the coordinate dimension of the point cloud, C is the feature dimension of the point cloud, each $N^{\prime }$ corresponds to a local region, and K represents the number of neighboring points in the center, as shown in Figure 8b.

In the CNN, convolution is the primary feature extractor, and the corresponding region is the (n,n) pixel region. In a 3D point cloud set, it is also necessary to find the sub-regions of the same structure, and the feature extractor of the corresponding region, which is used in the PNHM method, is PointNet. The PointNet layer mainly encodes the point cloud in many regions after grouping to obtain the feature vectors. It is necessary to input $N^{\prime }$ local point cloud regions of size $N^{\prime }*K*(d+C)$; each local region in the output has its features extracted via a process of encoding the center and the center field, and the output data size is $N^{\prime }*(d+C^{\prime })$. All points in a local region are first transformed into local coordinates using the center point as a reference, $x_{i}^{\left(j\right)}=x_{i}^{\left(j\right)}-{\hat{x}}_{i}^{\left(j\right)}$ where i=1,2,…,K and j=1,2,…d, with $\hat{x}$ representing the center point coordinates.

**Figure 9:**
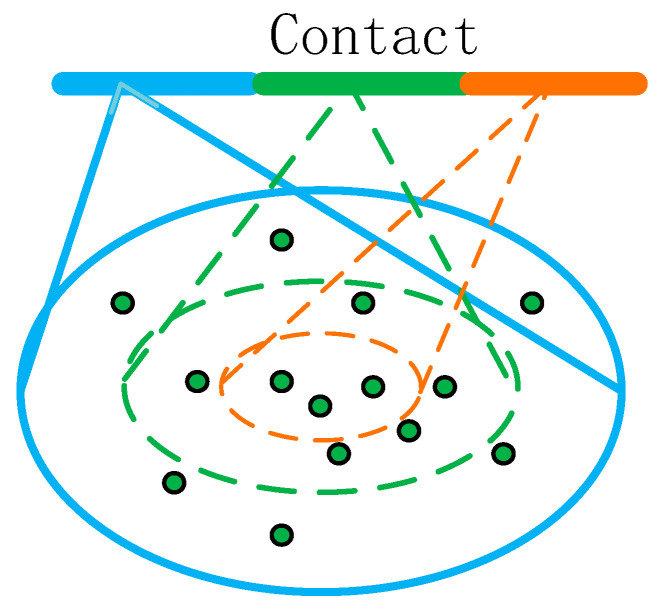
MSG processing of point clouds with inconsistent distribution.

The point clouds formed by four human body movements are characterized by irregular and sparse distribution, resulting in very few sampled point clouds in some regions if the same radius region is used in the above sampling process. In order to make the distribution of point cloud data in each region as uniform as possible, this paper uses the multi-scale grouping MSG (Muti-scale grouping) processing method, as shown in Figure 9. The blue, green and orange colors represent different radii of the 3D point cloud. For the same center point, three different radii are selected. The number of points in the neighborhood of the point cloud formed by the three different radii is inconsistent. The point clouds in the different radius regions are subjected to feature extraction using PointNet. Then, the features with different scales are connected to form the multi-scale features in order to obtain the features of the center point.

**Figure 10:**
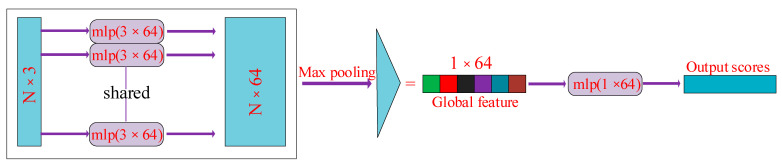
PointNet processing point cloud process.

Due to the large quantity of point cloud data and the disorder of point cloud data, the PoineNet neural network is required to process the point cloud data so that the results of different data arrangements are kept invariant. Because these data are the experimental number cloud data collected in 3D point text, there is a spatial relationship with the point cloud. The input to PointNet is a collection of 3D point cloud data in one frame, represented as data of N∗3, where N represents the number of point clouds and 3 represents the 3D spatial coordinates (x,y,z) of the point cloud. The input point cloud data are aligned via multiplication using the T−Net transformation matrix. T−Net is mainly composed of MLP. This ensures that the model is invariant to point cloud spatial transformations, allowing it to extract the same spatial features under different point cloud orders. Then, it is processed via MLP, which uses one-dimensional convolution. After the features are extracted from the point cloud data through multiple MLP processes, the features are aligned using T−Net. Max pooling is performed on each dimension of the point cloud features to obtain the final global features. Since the point cloud data does not need to be segmented, the global features obtained are predicted via MLP to obtain the final classification result. This process is shown in Figure 10.

**Figure 11:**
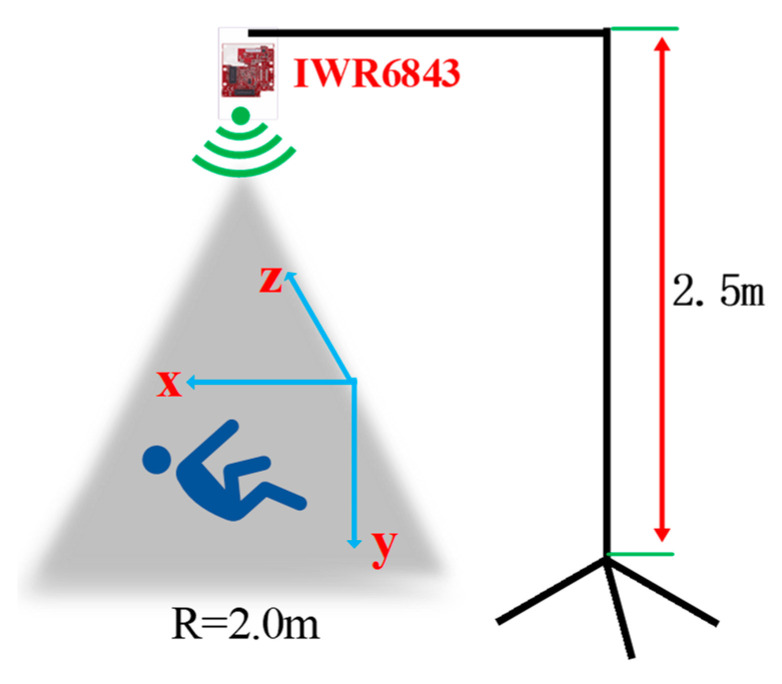
3D point cloud data acquisition scene when the radar is placed at a high altitude.

In order to make the experimental scenes diverse, this paper establishes a comparison scene domain for the four actions of falling, walking, standing to sit, and standing to squat. In some previous studies, radar was placed on the front or side of the human body to collect data. In this paper, the radar is also arranged parallel to the x−y plane and at a certain height from the ground. Based on the transmitting signal angle and coverage range of IWR6843, and considering the height of adults, the height of the radar from the ground is set at 2.5 m. In this paper, the 3D point cloud experiment of acquiring fall action is set within a radius of 2 m around the center area of the radar signal, as shown in Figure 11.

Because the 3D point cloud formed by human movements in this paper contains space–time information of multiple dimensions, the most basic information includes spatial coordinate information (x,y,z), and the original point cloud data also contain the experimental human body’s local speed and acceleration information. This information provides more dimensional detection methods for detecting movements such as falling and squatting. Because the height and speed of falls change quickly in these groups of basic movements, this paper identifies falling, walking, standing, and sitting according to the two threshold dimensions of height and speed.

## 5. Experimental Analysis 

**Figure 12:**
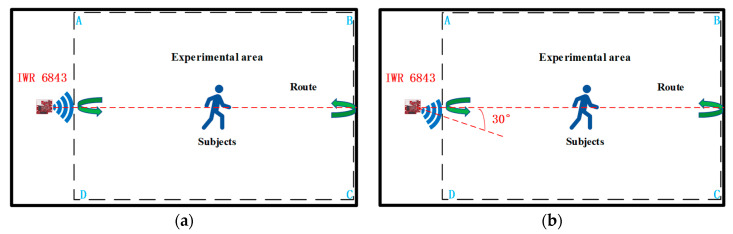
Experimental acquisition at two angles. (**a**)The subject is at an angle of 0° to the center of the radar. (**b**) The subject is at an angle of 30° to the center of the radar.

The equipment used in the experiment is TI’s millimeter-wave radar IWR6843 BOOST, which mainly works in the 60 GHz to 64 GHz band with a continuous bandwidth of 4 GHz. Its three parallel transmissions form the transmission subsystem. The four parallel channels form the receiving subsystem, the antenna’s transmitting period is 78/µs, and the frame period is set up according to the different qualities described above. The distance resolution is 5.49 cm, the maximum measured distance is 6.87 m, the maximum measured speed is 5.34 m, and the horizontal and elevation angles are between 60° and 120°. Figure 12a shows the process of experimental data acquisition from two angles in the laboratory scenario. The motion trajectory area of the experimental object in the laboratory designed in this paper is within a rectangle of 3 m × 4 m, and the motion trajectory area of the experimental object in the corridor scenario is within a rectangle of 2.5 m × 4 m. In order to ensure the diversity and reliability of the experimental data, the height, weight, age, and other factors of each experimental subject were selected to be different, with the height ranging from 155 cm to 181 cm, the weight ranging from 45 kg to 70 kg, and the age ranging from 22 years old to 28 years old. This ensured the high quality and diversity of the point cloud data as much as possible. After completing 900 sets of point cloud data collection in one month, 75% of the point cloud data set was used as the training data set, 25% of the point cloud data were used as the test data set, and the training data set and the test data set were used in a ratio of 3:1. We considered that the radar signals emitted from the millimeter-wave radar IWR6843 have specific elevation and horizontal angles. Combined with wise aging, health detection, and the height of the general public, this paper also considered background practical factors, as well as the critical point of the height of ordinary people. In addition to the most important factor, background practical factors included the need to obtain the most complete 3D point cloud data possible to move each experimental object through the radar signal area, producing a complete 64-point cloud. The radar height was arranged to be 1.5 m from the ground; the duration of each movement was 50 s, for a total of 1000 frames. We set up this experiment in the laboratory and the corridor under the scene, adding radar signal center areas of 0° and 30° of the scene to the data collection. In this paper, the number of point clouds in each frame was only 64 to ensure the high quality of the point cloud data. This also establish the experimental object’s trajectory and the center of the radar-transmitting signals, the maximum difference between them of 30°, and the center of the radar signal, as shown in Figure 12a,b.

In this paper, we introduced earlier the idea that the number of frames transmitted per second is set for each action according to the different duration of each action. For PNHM neural networks, the data frames of the point cloud represent how many times the point cloud of the human body action was assessed. The sign of the point cloud contains the spatial and temporal information of the human body action, the frequency and speed of the body movement, the acceleration of localized parts, the magnitude of information, etc. If the data frame input into the PNHM method is too small, it cannot fully present the various human body action characteristics after training. Suppose the data frame inputs to the PNHM method are small. In that case, they cannot fully present the characteristics of various human actions, and the accuracy obtained after training cannot be optimized. When the data frames input to the PNHM method are too long, not only will the training time be very long, but the sample data for testing will also be reduced.

**Figure 13:**
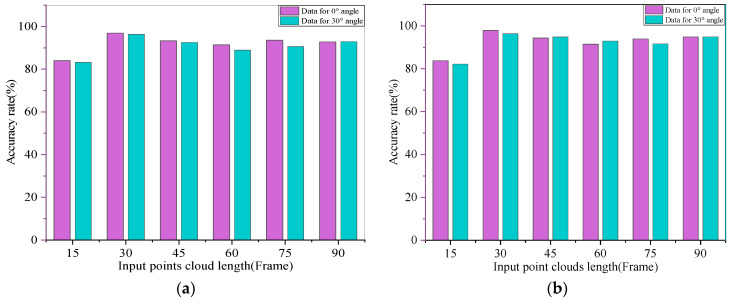
Accuracy of different data frames in two experimental scenarios. (**a**) Accuracy under different data frames in the lab; (**b**) accuracy at different data frames in the corridor.

Consecutive point cloud frames contain more spatial and temporal patterns of human walking. A single radar device is always used for point cloud data acquisition in this paper in order to obtain a better training effect. Then, according to the frame frequency set by the cycle of different actions before, all the action point cloud data frames are input at 15 frames per superimposition to obtain the accuracy rate under different frames. The results show that the best performance in terms of accuracy in the laboratory scene and the corridor scene was when the data were taken as one input at 30 frames. As such, this paper took all the action point cloud data as a sub-input to the PNHM method with 30 frames of point cloud per cut. Attributes from multiple dimensions of the point cloud data were input for training, including coordinate information, signal-to-noise ratio, altitude and velocity thresholds in 3D, and the accuracies under different point cloud frame inputs at two angles under the corridor and in the laboratory environment are shown in Figure 13a,b. 

**Figure 14:**
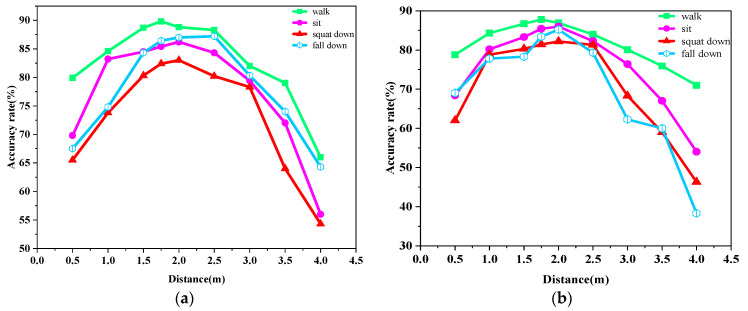
The accuracy rates of walking, standing to sitting, and standing to squat at two angles in the laboratory scene. (**a**) Accuracy at different distances and movements at 0° angle in the lab; (**b**) accuracy of different distances and movements at 30° angle in the lab.

Considering a real-life scenario, the angle between the human body movement trajectory and the signal center of the radar device may take random values. Therefore, in this paper, the laboratory placed the radar on the front or side of the experimental object for measurement and established the recognition rate of various human motions under two angles, as shown in Figure 14a,b. We found that the action recognition rate under either angle was unsatisfactory within 0.5 m. Within 2.5 m, the recognition rate when the trajectory of the experimental object was at an angle of 0° to the center of the radar signal was slightly higher than the recognition rate when the trajectory of the experimental object was at an angle of 30° to the center of the radar signal. The recognition rates of all three actions showed a rapidly decreasing trend after the human body was more than 2.5 m away from the radar. In this scenario, the recognition rate was still lower compared to the action of walking because the standing-to-sitting and standing-to-squatting actions presented very similar point clouds and did not have height and velocity thresholds as attributes for input.

**Figure 15:**
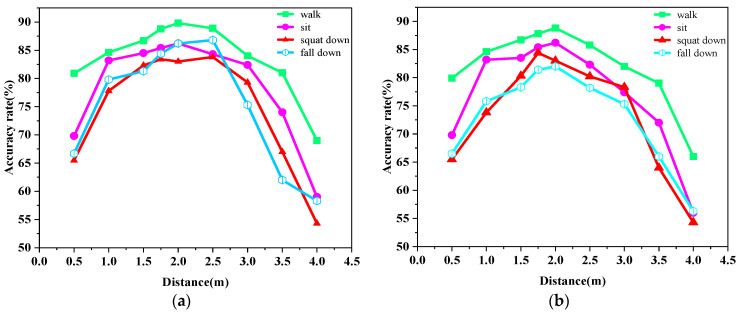
The accuracy rate of walking, standing to sitting, and standing to squat at two angles in the corridor scenario. (**a**) Accuracy of different distances and movements at 0° angle in a corridor; (**b**) accuracy of different distances and movements at 0° angle in the corridor.

The accuracy rates of the four actions from the corridor scene at different angles are shown in Figure 15a,b. In the corridor scene, the experimental area was narrower, which produced more clutter. After removing the noise, the point cloud data obtained was sparser, and overall the human action recognition accuracy in the corridor scene was lower compared to the human action recognition accuracy in the lab scene at the same distance. 

In this paper, due to the three-dimensional point cloud data formed by the human body, very sparse action and scattered point cloud mapping of the picture produced a large amount of redundant data and significantly consumed the network bandwidth, or used image vision for recognition. However, excessively sparse point cloud data in the four kinds of action presented in the picture makes it difficult to perform high-precision recognition.

**Figure 16:**
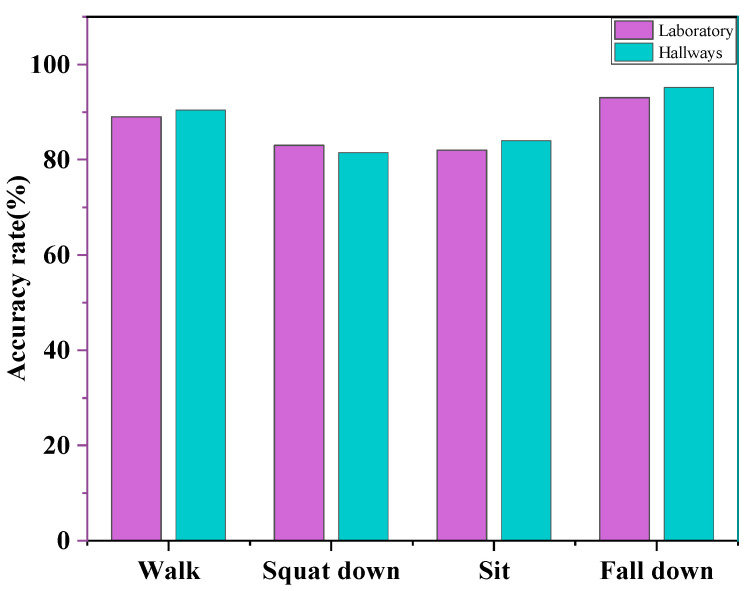
Recognition rate of various behavioral actions when the radar is placed at 2.5 m from the floor.

After analyzing the characteristics of the point cloud data in this paper, the point cloud data for speed, height, and other attributes were able reflect the characteristics of each part of the human body well to produce a variety of actions. Many frames of the continuous point cloud is more reflective of the regularity of each action, and therefore with the help of the point cloud speed and height threshold changes in the recognition could be made. In the literature [22], some tests of speed and height were conducted for squatting, falling, sitting, and walking, and the results showed that squatting and sitting have some similarities. The test data of each movement comprised 20 frames, and the results showed that the velocity values of squatting movement were distributed between 0 m/s and 1.3 m/s, and the velocity values of sitting down movement were distributed between 0 m/s and 1 m/s. The dynamic data for the falling action comprised 30 frames. The test was conducted because some personnel would lift their feet, resulting in some points with negative velocity values of the data. The velocity values for the falling action were distributed between −0.5 and 1.8 m/s, and the velocity values for the walking action were distributed between −1.3 m/s and 1.2 m/s. The four actions are generally very different, but the same actions performed for different individuals have significant similarities. The design of this paper placed the radar at 2.5 m from the ground, and the recognition rates of falling, walking, and standing to sit in the laboratory under two angular scene domains are shown in Figure 16. In this scenario, the height and speed threshold are the core attributes for recognizing various human behaviors. Due to the significant differences in actions, such as walking and falling, the recognition rate is lower in recognizing the three actions of falling, sitting to standing, and squatting to standing. There are approximations to the speed and height thresholds for these three types of actions.

**Figure 17:**
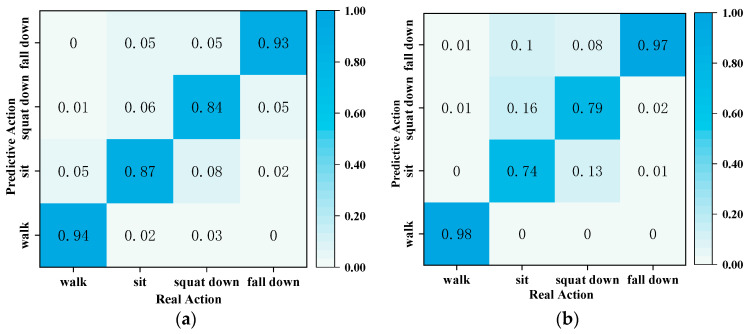
Comparison of test results under the two methods. (**a**) Method based on PNHM neural network; (**b**) method based on speed and height thresholds.

In this paper, the PNHM neural network model and the method using thresholding were again utilized to recognize and predict the actions of the point cloud, and a comparison of the two types of methods is shown in Figure 17a,b. Figure 17a shows sample data from the PNHM method for the recognition method, in which the prediction accuracy of walking, sitting, squatting, and falling is 94%, 87%, 84%, and 93%, and its average recognition correctness is 89.5%. In Figure 17b, all of its sample data are recognized based on the method of speed and height thresholds, in which the prediction accuracy of walking, sitting down, squatting, and falling is 98%, 74%, 79%, and 97%. Its average recognition correctness rate is 87% due to the differences in height and weight of each experimental subject in the sample data, and squatting and sitting down have some similarities in speed and height thresholds. So, the recognition is lower compared to the other two actions.

**Figure 18:**
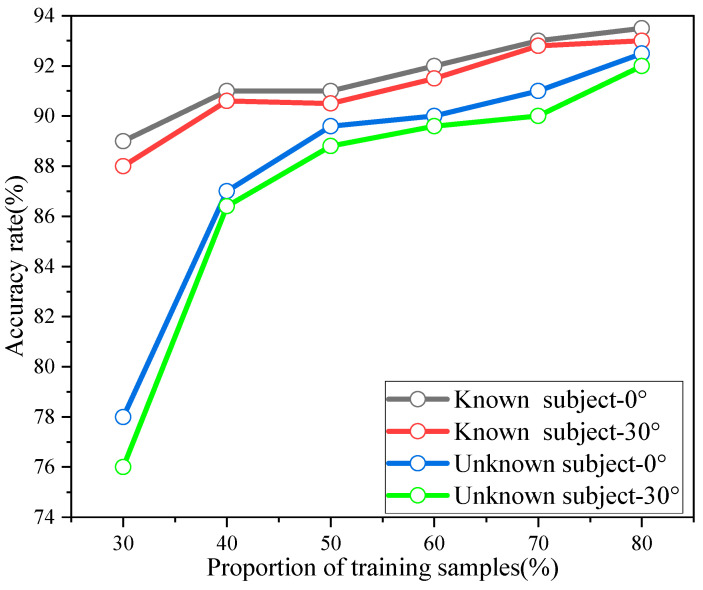
Recognition accuracy of known and unknown individuals under different training sets.

In order to illustrate the advantages of the proposed method PNHM in this paper for point cloud recognition, the point cloud data were tested using different training data sets and test data sets share. The test scheme saw the unknown experimental subjects predicted using the point cloud data of the known experimental subjects, and the data under two experimental perspectives of the laboratory and the corridor were tested according to the training data set and the test data set with different shares. Figure 18 shows the accuracy of known and unknown experimental subjects under different training sets. The black and red lines are the recognition accuracies of the point cloud data of the known experimental objects, which are the point cloud data collected when the experimental objects and the radar center are at an angle of 0° and an angle of 30°, respectively. The prediction results for approximately 75% of the training samples in this paper had accuracies set to make the ratio of training samples and test samples 3:1. The blue and green lines are the accuracies at different percentages of training and testing data sets. The blue and green lines are the recognition accuracy of the point cloud data of the unknown experimental object, which represents the point cloud data collected when the unknown experimental object and the radar center are at angle of 0° and an angle of 30°. It can be concluded from Figure 18 that the point cloud data accuracy of the unknown experimental object is greatly affected by the training data set share; with the training test set share of 75%, its recognition accuracy and the recognition accuracy of the known experimental object are not greatly different. Comparing the same training set test, the known experimental object’s prediction of the recognition accuracy was higher than that of the unknown experimental object’s prediction accuracy. However, the recognition accuracy of the unknown experimental objects could also reach about 87% in the case of 40% of the training test set; in addition, with the increase in the proportion of the training test set, its recognition accuracy continued to gradually increase. The robustness of the PNHM method for recognizing point clouds could thus be verified. 

In order to compare the effectiveness of the PNHM method, this paper compare three mainstream point cloud recognition algorithms, CNN, LSTM, and PointNet+LSTM, with recent research and compare the various neural network methods of recognition accuracy as an indicator, as shown in Table 1. This paper’s point cloud data, cut into a burst of frames, is input to the following algorithms for recognition. PointNet appears several times in the set abstraction part of the PNHM method and the classification module, here Laboratory-0° and Corridor-0° represent the point cloud data when the experimental subject and the radar center are at an angle of 0° in the laboratory and down the corridor. Laboratory-30° and Corridor-30° represent the point cloud data when the experimental subject and the radar center are at an angle of 30° in the laboratory and down the corridor. The results in the table show that the recognition accuracy under the CNN or LSTM neural network method alone is lower than that under the PointNet+LSTM neural network, and that a combination of spatio-temporal feature extraction using the network PointNet+LSTM can improve the feature recognition accuracy of the point cloud data of the human body movements. The method PNHM designed in this paper is based precisely on the PointNet++ neural network, fusing local and global point cloud data features, and its recognition accuracy is superior to the other three mainstream point cloud recognition algorithms in various scenarios.

## 6. Conclusions

This paper used a human action recognition system (PNHM) based on the PointNet++ neural network to recognize human basic actions via the millimeter-wave radar 3D target point cloud formed by human basic actions for classification. First, the dynamic point cloud data formed by human actions were collected by making the FMCW millimeter-wave radar IWR6843 device face the experimenter and making the center of the emitted signal of the IWR6843 radar device at an angle of 0° and 30° to the movement trajectory of the experimenter. The experiments were conducted in two scenarios, namely, in the laboratory and in the corridor, respectively. Then, a neural network recognition system for the dynamic point cloud of human movement was designed. After denoising and clustering the obtained dynamic point cloud data, the dynamic point cloud data was cut into static point cloud data frames and input to the system using a similar processing method to convert to static point cloud data, and feature extraction was performed for the temporal and spatial attributes of the 3D dynamic point cloud of the human body movements. Finally, the PNHM method trained and classified the obtained point cloud attribute information to recognize the human actions corresponding to the 3D point cloud data, and this neural network recognition system performed well in the recognition accuracy of human actions. Under the same angle, the system’s recognition accuracy of basic human actions in the two experimental environments did not differ much. However, the recognition rate of human actions in the experimental scenario where the signal center of the radar equipment was facing at a 0° angle to the experimenter’s motion track was higher than that in the scenario where the signal center area of the radar equipment was at a 30° angle to the experimenter’s motion track. Compared with several mainstream point cloud recognition methods, this method had a higher recognition rate for human action point clouds in all five scenarios.

Future work should be carried out due to the following issues: (1) In this paper, due to the experimental design being only for the action recognition of a single experimental subject, relevant research was not carried out for action recognition with the coexistence of multiple people in a scene. (2) The experimental data acquisition was only carried out under the angle of 0° and 30° between the signal center of the radar equipment and the movement trajectory of the experimenter, and experiments were not carried out under more angles. Hence, the robustness of this experiment needs to be improved.

## Figures and Tables

**Table 1:** Comparison of recognition accuracy between other point cloud recognition algorithms and PNHM methods for human actions in this paper.

Method	CNN	LSTM	PointNet+LSTM	Ours
Laboratory-0°	81.3%	84.4%	86.8%	90.4%
Corridor-0°	82.2%	84.7%	86.9%	88.6%
Laboratory-30°	81.6%	78.6%	80.6%	85.7%
Corridor-30°	76.2%	80.6%	83.8%	86.2%

## Data Availability

The data presented in this study are available on request from the corresponding author. The data are not publicly available due to the team has not fully completed the project, the data is not disclosed for the time being.
